# A p53-Dependent Response Limits Epidermal Stem Cell Functionality and Organismal Size in Mice with Short Telomeres

**DOI:** 10.1371/journal.pone.0004934

**Published:** 2009-03-19

**Authors:** Ignacio Flores, Maria A. Blasco

**Affiliations:** Telomeres and Telomerase Group, Molecular Oncology Program, Spanish National Cancer Centre (CNIO), Madrid, Spain; Ordway Research Institute, United States of America

## Abstract

Telomere maintenance is essential to ensure proper size and function of organs with a high turnover. In particular, a dwarf phenotype as well as phenotypes associated to premature loss of tissue regeneration, including the skin (hair loss, hair graying, decreased wound healing), are found in mice deficient for telomerase, the enzyme responsible for maintaining telomere length. Coincidental with the appearance of these phenotypes, p53 is found activated in several tissues from these mice, where is thought to trigger cellular senescence and/or apoptotic responses. Here, we show that p53 abrogation rescues both the small size phenotype and restitutes the functionality of epidermal stem cells (ESC) of telomerase-deficient mice with dysfunctional telomeres. In particular, p53 ablation restores hair growth, skin renewal and wound healing responses upon mitogenic induction, as well as rescues ESCmobilization defects *in vivo* and defective ESC clonogenic activity *in vitro*. This recovery of ESC functions is accompanied by a downregulation of senescence markers and an increased proliferation in the skin and kidney of telomerase-deficient mice with critically short telomeres without changes in apoptosis rates. Together, these findings indicate the existence of a p53-dependent senescence response acting on stem/progenitor cells with dysfunctional telomeres that is actively limiting their contribution to tissue regeneration, thereby impinging on tissue fitness.

## Introduction

The mechanisms that dictate body size and frailty of organisms are still poorly understood. One determinant of both biological traits is proposed to be the number of functional stem cells within tissues as stem cells are crucial in generating, regenerating and maintaining tissues throughout organism life span [1], [2]. In support of this notion, a number of signaling pathways have been described that when activated result both in increased organ size and expanded stem/progenitor cell pools, such as components of the IGF-1 and the Hippo/YAP pathways [3]–[5]. In contrast, overall body size reduction, stem cell dysfunction and organ failure are hallmarks of the elderly [6]. Similarly, mice showing growth retardation and premature aging phenotypes also have decreased numbers of functional stem cells within tissues [7]. Together, these findings suggest that occurrence of reduced organismal size and tissue dysfunction are likely linked to the exhaustion of functional stem cell pools, in agreement with a “rate-of-living” theory of aging specifically acting on stem cells [8].

Telomere shortening has been shown to affect both organismal size and stem cell functionality in the context of telomerase-deficient mice [9]. Telomeres are ribonucleoprotein complexes at the ends of eukaryotic chromosomes that have an essential role in protecting chromosome ends for DNA repair and degrading activities [10]–[13]. A minimal number of TTAGGG tandem repeats and the integrity of a six-protein complex known as shelterin are required to ensure telomere protection [14]. If telomere function is compromised, i.e., by altering shelterin components or by severe telomere erosion, a robust DNA damage response is activated leading to cellular senescence and/or apoptotic responses [15], [16]. Interestingly, an age-dependent accumulation cells with damaged telomeres (ie, cells showing co-localization of gamma-H2AX foci and telomeres) has been reported in primates, suggesting that telomere dysfunction may act as a chronological clock [17]. In this regard, telomere shortening occurs associated to mouse and human aging and has been proposed to be rate-limiting for organismal life span [18]–[20]. Importantly, telomere shortening associated with aging is observed both at stem cell and differentiated compartments in humans and mice [18], [19], opening the possibility that telomere erosion with age may be responsible, at least in part, for the decline in stem cell functionality associated to the aging process [20], [21]. This notion is supported by telomerase-deficient mice with short telomeres, which show severely compromised epidermal stem cell functionality with increasing mouse generations [9]. Likewise, severe telomere attrition in these mice leads to the occurrence of a dwarf phenotype [9].

The tumor suppressor protein p53 is activated and mediates the cellular response to various types of DNA damage, including telomere dysfunction [22], [23]. In particular, abrogation of p53 rescues male germ cell depletion in telomerase-deficient mice with short telomeres, suggesting that p53 senses telomere damage in stem/progenitor cell populations and leads to massive germ cell apoptosis [24], [25]. In turn, p53 abrogation also impairs the tumor suppressor activity of short telomeres leading to increased tumorigenesis in telomerase deficient mice simultaneously lacking p53 [25], [26].

Here, we set to address the role of p53 in both organismal size determination and in epidermal stem cell (ESC) behavior (ESC mobilization and clonogenic activity, wound healing, hair growth, skin regeneration) in mice with dysfunctional telomeres by generating doubly deficient mice for telomerase and p53, *Terc^−/−^p53^−/−^*. We found that p53 ablation rescues the mitogen induced hair and skin growth responses, at the same time that it corrects ESC activation and the dwarf phenotype of late-generation telomerase deficient mice. All together, these findings support the notion that p53 plays a central role in limiting the functionality of epidermal stem cells as well as in restricting organismal size in mice with dysfunctional telomeres.

## Results and Discussion

### p53 abrogation rescues epidermal and hair growth defects in telomerase-deficient mice with short telomeres

**Figure 1:**
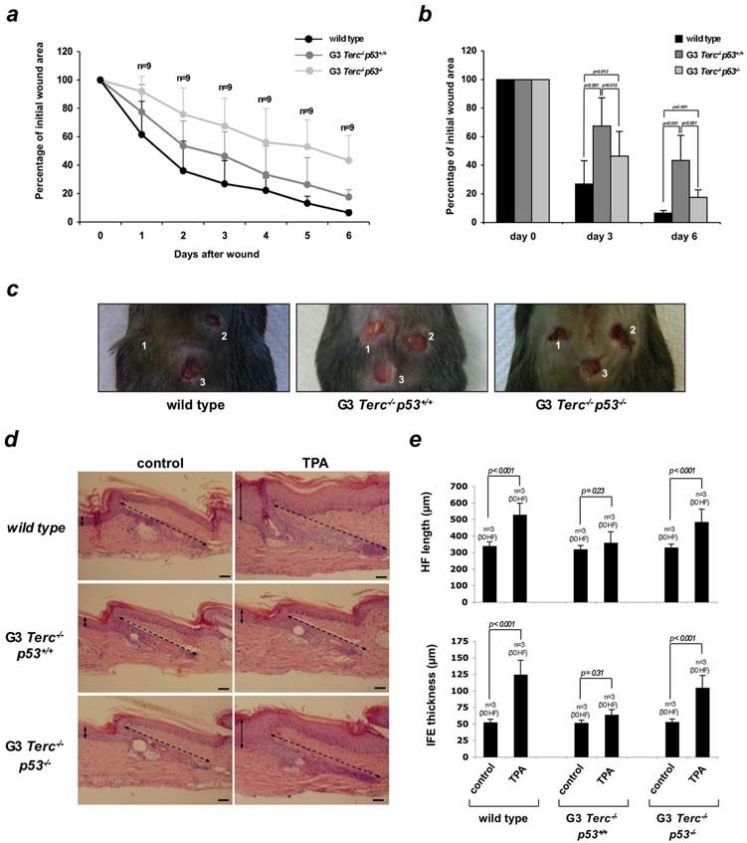
p53 deletion rescues epidermal and hair growth responses in late-generation telomerase-deficient mice. (A) Wound healing of wild-type, G3 *Terc^−/−^* and G3 *Terc^−/−^p53^−/−^* skin. (B) Percentage of initial wound area left at the indicated days after the punch was created. Three wounds were created per mouse, and the total number of wounds per genotype is indicated in the graph (n). (C) Statistical analysis showing that the healing rate is significantly different between genotypes is also shown (p-values). Bottom: Representative images of wounds 4 days after the first wound was made (1, 2 and 3 in the figure correspond to the first, second and third wound at 4, 2 and 0 days, respectively, after the first wound was created). (D) Representative tail-skin sections from wild-type, G3 *Terc^−/−^* and G3 *Terc^−/−^p53^−/−^* littermates before and after TPA-treatment. Continuous black double-point arrows mark inter-follicular (IFE) thickness. Black dashes double-point arrows mark hair follicle (HF) length. (E) Quantification of the HF length (bottom) and IFE thickness (top). Histomorphometry was performed in three mice of each genotype (n), quantifying 30 hair follicles (HF) per group. Note the increased HF length and epidermis thickness in TPA-treated wild-type and G3 *Terc^−/−^p53^−/−^* mice, compared with a defective response in TPA-treated G3 *Terc^−/−^* animals. Scale bar, 50 µm.

The skin is one of the tissues where the pro-aging effects of short telomeres are more clearly visible in the context of telomerase-deficient mice [27]. In particular third generation (G3) *Terc^−/−^* mice show a delay in wound closure and a stunted hair growth response after mitogen induction [9], [27]. These skin defects are further aggravated at old ages leading to severe premature alopecia and hair graying in late generation *Terc^−/−^* [27]. Here, we set to address whether p53 activation in response to short telomeres contributes to defective skin and hair regeneration by limiting ESC functionality. To this end, we generated increasing generations of mice doubly deficient for Terc and p53 reaching third generation (G3) *Terc^−/−^p53^−/−^* mice, as well as the corresponding G3 *Terc^−/−^p53^+/+^* littermate controls (see Methods for breeding strategies). First, we assessed the role of p53 in the impaired wound healing response associated to critically short telomeres [27] To this end, three consecutive punch biopsies were performed on dorsal skin of age-matched (2-months old) G3 *Terc^−/−^p53^−/−^* and G3 *Terc^−/−^p53^+/+^* littermates, as well as wild-type controls (Methods). The rate of wound healing was monitored as the percentage of the initial wound area left open at different times after the wound was made (Methods). One wound was created 2 days after the other and the animals were killed 6 days after the last wound was made. As previously reported for middle-age telomerase-deficient mice, young G3 *Terc^−/−^p53^+/+^* mice exhibited a delay in wound closure (Fig. 1a–c), with average wound areas at day 3 of 26% and 67% for wild type mice and G3 *Terc^−/−^p53^+/+^* mice, respectively (p = 0.013) and 6% and 43% for wild type mice and G3 *Terc^−/−^p53^+/+^* mice, respectively at day 6 (p<0.001; Fig. 1a–c). Interestingly, we observed a partial recovery to normal wound healing rates in the case of G3 *Terc^−/−^p53^−/−^*. At day 3, only 46% of the initial wound area persists, which is further decreased to 17% three days later (Fig. 1a–c). Taken together, these results indicate that the skin of G3 *Terc^−/−^p53^−/−^* mice presents a faster wound-healing rate than that of the corresponding G3 *Terc^−/−^p53^+/+^* littermates, supporting an inhibitory effect of p53 in the wound healing process in mice with short telomeres.

We next assessed the role of p53 in the impaired hair growth response associated to critically short telomeres. Hair follicle (HF) length and inter-follicular (IFE) skin thickness were not significantly increased in G3 *Terc^−/−^p53^+/+^* mice upon TPA treatment (Fig. 1d,e), in agreement with a defective ESC functionality in these mice [9]. Strikingly, p53 abrogation rescued the defective hair growth response as well as the epidermal hyperplastic response in G3 *Terc^−/−^p53^−/−^* mice to a similar extend than that of wild-type mice, as indicated by significantly increased HF length and IFE thickness in response to TPA treatment compared to control “resting” non-treated skin (Fig. 1d,e). This finding indicates that p53 deletion can restore both hair growth and epidermal defects in mice with short telomeres to wild-type levels, suggesting the existence of a p53-dependent checkpoint that is limiting the entry of hair follicles in the anagen (growing) phase of the hair cycle in the presence of critically short telomeres. Unfortunately, early death of G3 *Terc^−/−^p53^−/−^* cohorts due to an early onset of lymphomas and sarcomas associated to p53-deficiency (ie., all mice were dead with in the first 40 weeks of life; not shown) prevented the analysis of the role of p53 in additional skin defects associated to critically short telomeres, such as hair graying and alopecia [27], [28].

### p53 ablation restores tissue mobilization of ESC harbouring dysfunctional telomeres

**Figure 2:**
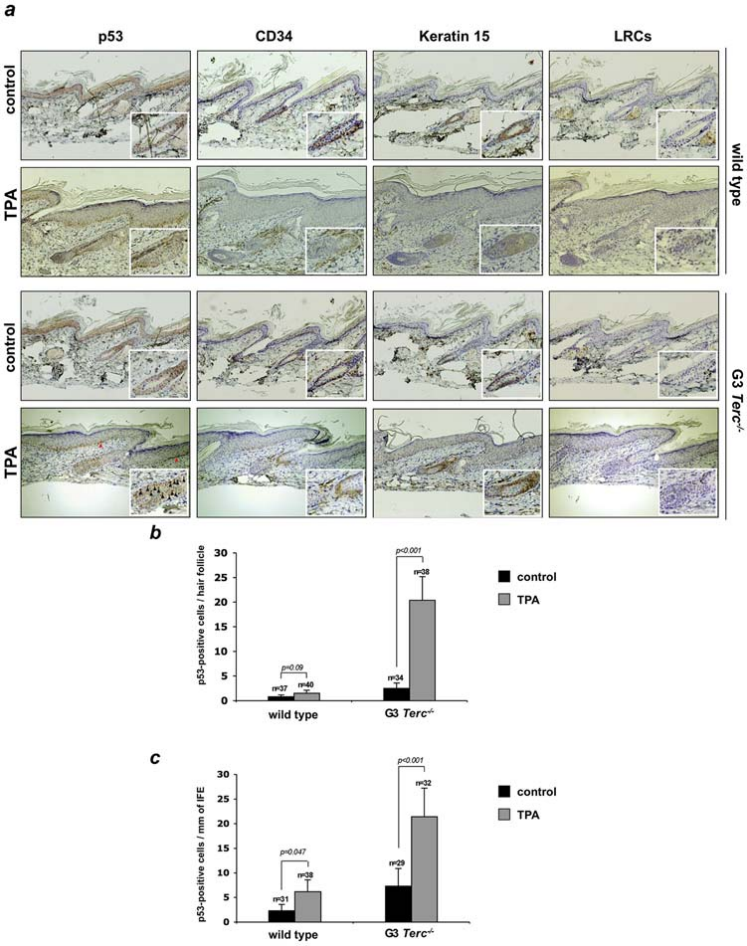
p53^high^ positive cells in late-generation telomerase-deficient mice are found preferentially at the hair bulge after TPA stimulation. (A) Immunohistochemistry was performed with anti-p53 antibodies in tail skin for wild type and G3 *Terc^−/−^* mice before and after TPA treatment. Positive cells with a clear staining for p53 were found scattered within the bulge, the hair follicle stem cell compartment, and their close proximity in G3 *Terc^−/−^* animals upon TPA stimulation as well as in patches within the basal layer (see red arrows points, left panels). Hair bulge compartment has been defined by CD34 and keratin15 positive staining in addition to accumulation of LRCs. Inserts: high magnification images showing the bulge area. Black arrows points to p53^high^ positive cells. Number of p53 positive cells at the hair follicle (B) and interfollicular epidermis (IFE) (C) in the conditions and genotypes shown in (A).

**Figure 3:**
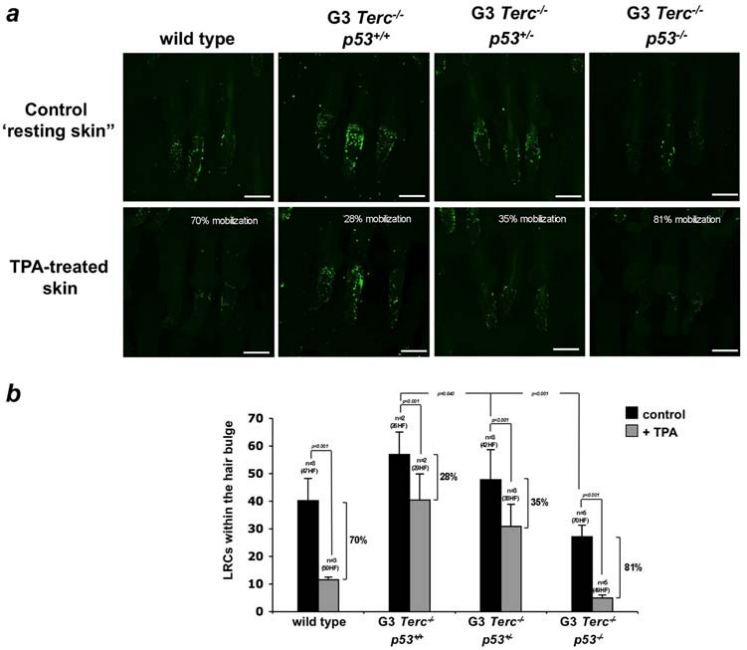
p53 loss rescues the hair follicle stem cell activation response in late-generation telomerase-deficient mice. (A and B) To assay for LRC activation, G3 *Terc^−/−^* and G3 *Terc^−/−^p53^−/−^* littermates were injected with repetitive pulses of BrdU and, after 69 days, whole mounts of tail epidermis were collected from untreated and TPA-treated mice and stained with a BrdU antibody. (A) Representative confocal micrographs of tail follicles from G3 *Terc^−/−^* , G3 *Terc^−/−^p53^+/−^* and G3 *Terc^−/−^p53^−/−^* mice stained for BrdU (green) before (control) and after (TPA) TPA treatment. (B) Number of LRCs in the conditions and genotypes shown in (A). LRCs numbers were calculated in (n) number of mice of each genotype, quantifying at least 26 hair follicles (HF) per group. Note a higher percentage of LRCs activation in TPA-treated G3 *Terc^−/−^p53^−/−^* and G3 *Terc^−/−^p53^+/−^* mice compared with G3 *Terc^−/−^* mice. For comparison purposes, LRCs activation in TPA-treated G3 *Terc^−/−^p53^−/−^* mice reached 81% (81% decrease in LCRs following TPA treatment compared to resting non-treated controls), which is similar to that previously described for wild-type ESC of the same genetic background (70% activation; Flores et al., 2005). LCR activation is calculated as percentage LCR decrease at the hair bulge following TPA treatment compared to the resting-skin (control). Scale bar, 80 µm.

Considering the important contribution of stem cells to organ development and tissue fitness, we next addressed whether the ability of ESC to regenerate skin and the hair was limited by p53 in the presence of short/dysfunctional telomeres. First, we examined whether p53 accumulates in skin cells from late generation telomerase-deficient mice. As shown in Fig. 2a, we found a significant number of p53-positive cells in the skin of G3 *Terc^−/−^* mice after TPA treatment, which were rarely observed in the corresponding age-matched TPA-treated wild-type controls (Fig. 2a, see Fig. 2b,c for quantification). Interestingly, the majority of p53-positive cells were located at the hair bulge and their close proximity, as well as we detected patches of p53-positive cells at the basal layer of the epidermis (Fig. 2a–c), known locations for stem/progenitor cells in mouse epidermis [29], [30] , suggesting that p53 is activated in stem/progenitor cells in the presence of short telomeres. Next, to visualize and study the behavior of epidermal stem cells, we used a labeling technique previously shown to mark self-renewing and multi-potent epidermal cells, the so-called “label retaining cells” (LRCs) (Methods and Methods S1) [31], [32]. Confocal microscopy of skin whole-mounts revealed that LRCs accumulated at the bulge area of the hair follicle in all the examined genotypes, in agreement with the known location of the hair follicle stem cell niche [9], [33]–[36] (Fig. 3a, see also Fig 2). Interestingly, in resting-skin conditions both G3 *Terc^−/−^p53^−/−^* and G3 *Terc^−/−^p53^+/−^* hair follicles contained significantly less LRCs compared to G3 *Terc^−/−^p53^+/+^* follicles, indicating a higher rate of label disappearance in G3 *Terc^−/−^* cells, which is associated with decreasing p53 gene levels (Fig. 3a,b). This finding suggests that p53 gene dosage is rate limiting in detecting telomere damage and that G3 *Terc^−/−^p53^−/−^* and G3 *Terc^−/−^p53^+/−^* keratinocytes are more prone to exit the stem cell niche and to proliferate than G3 *Terc^−/−^p53^+/+^* keratinocytes. Despite the above differences in LRC numbers in resting conditions, we have not detected significant alterations on the hair cycle stage between G3 *Terc^−/−^p53^−/−^*, G3 *Terc^−/−^p53^+/−^* and G3 *Terc^−/−^p53^+/+^* animals (Fig 1b,c and data not shown). To specifically assess whether ESC activation differs between the different genotypes, the skin was treated with TPA as previously described (Methods). Upon TPA treatment, only 28% of G3 *Terc^−/−^p53^+/+^* LRCs were activated (calculated as percentage LCR decrease at the hair bulge following TPA treatment compared to the resting-skin control), confirming a defective ESC activation in *Terc^−/−^* mice with short telomeres (Fig. 2a,b) [9]. Furthermore, in agreement with a defective mobilization of G3 *Terc^−/−^p53^+/+^* ESC, scattered LRC are still detectable within the infundibulum and basal layer of the IFE, which could partially explain why the infundibulum and the IFE did not thicken following TPA treatment (Fig. 2a). In contrast, up to 35% of LRCs from TPA-treated G3 *Terc^−/−^p53^+/−^* were activated. This mobilization response was even more pronounced in the case of G3 *Terc^−/−^p53^−/−^* mice reaching a similar activation level to that previously described for wild-type ESC of the same genetic background (70% activation; Fig. 3a,b) [9]. Concomitant with the restoration of the ESC activation response, the absence of p53 was accompanied by a clear enlargement of the transient amplifying compartments of G3 *Terc^−/−^p53^−/−^* mice (Fig. 3a,b). These results indicate that p53 dosage influences ESC behavior of mice harboring critically short telomeres, leading to normal activation responses when p53 is absent.

### p53 ablation increases the clonogenic activity of ESC with short telomeres

**Figure 4:**
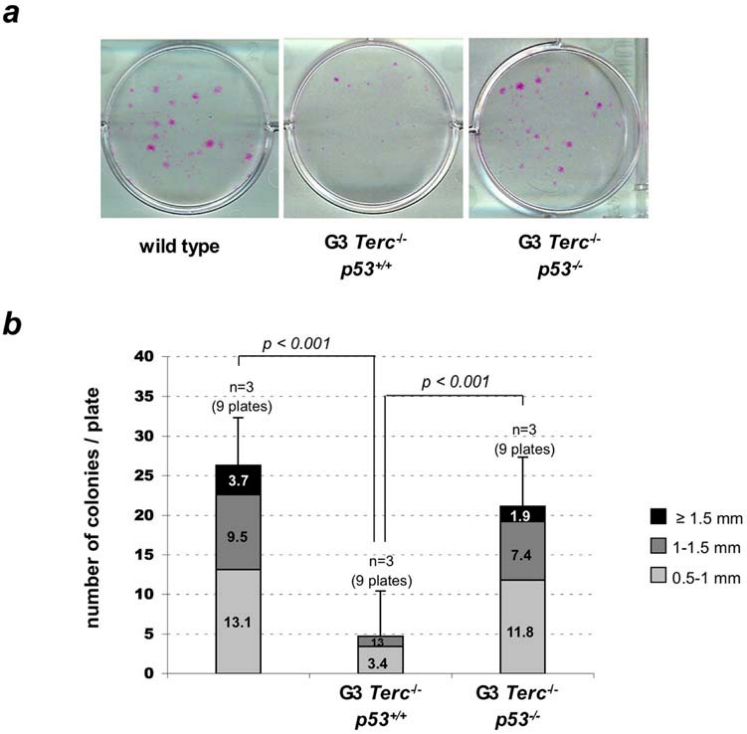
p53 abrogation rescues the proliferation potential of epidermal stem cells in late-generation telomerase-deficient mice. (A) Representative images of number and size of macroscopic colonies obtained from isolated keratinocytes from wild-type, G3 *Terc^−/−^* and G3 *Terc^−/−^p53^−/−^* littermates. Note more abundant and larger colonies in wild-type and G3 *Terc^−/−^p53^−/−^* compared with G3 *Terc^−/−^*. Individual colonies in this assay have been proposed to derive from single ESC [37]. (B) Quantification of size and number of macroscopic colonies obtained from isolated keratinocytes from the indicated genotype purified from a total of 3 independent 2-days old mice and cultured for 1 week on J2-3T3 mitomycin C-treated feeder fibroblast.

We previously described that ESC derived from late generation telomerase-deficient mice with short telomeres have a decreased proliferation potential when cultured *ex vivo* using the so-called clonogenic assays [9] (Methods and Methods S1). The number and size of colonies in these assays is proposed to reflect on the proliferation potential of individual ESCs [37]. Furthermore, individual colonies in clonogenic assays have been proposed to derive from single ESCs [37]. Here, we set to address whether absence of p53 rescues the decreased clonogenic activity of ESC with short telomeres. To this end, we performed clonogenic assays with newborn keratinocytes isolated from G3 *Terc^−/−^p53^−/−^* and G3 *Terc^−/−^p53^+/+^* mice, as well as the corresponding wild-type controls (Methods). Interestingly, G3 *Terc^−/−^p53^−/−^* cells showed a similar clonogenic potential to that of wild-type cells, while G3 *Terc^−/−^p53^+/+^* cells were severely affected (see number and size of colonies in Fig. 4a,b), indicating that p53 abrogation fully rescues the proliferation potential of ESCs with short/dysfunctional telomeres *ex vivo*. All together, these results indicate that p53 ablation rescues both the cell-autonomous ESC proliferation defects (Fig. 4), as well as the *in vivo* ESC “mobilization” defects (Fig. 3a,b) in mice with critically short telomeres.

### p53 abrogation rescues the “small-size” phenotype of mice with critically short telomeres

**Figure 5:**
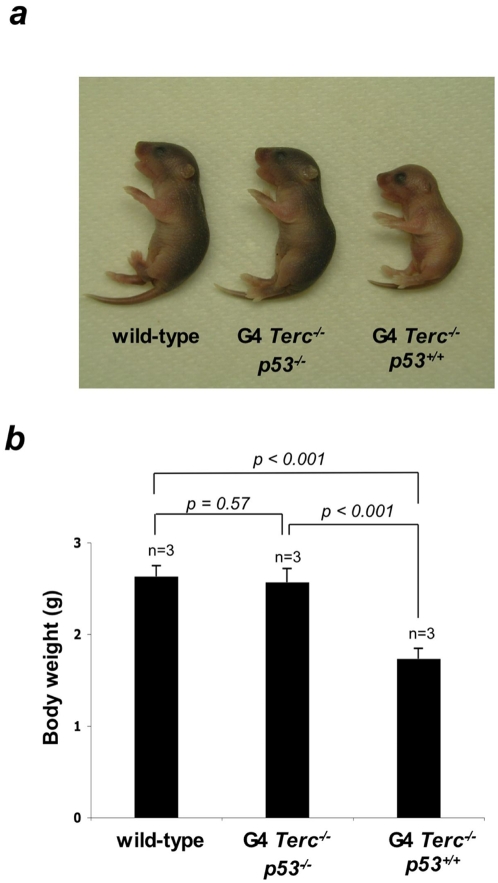
p53 abrogation rescues the small body-size phenotype in late-generation telomerase-deficient mice. (A) Representative images of wild-type, G4 *Terc^−/−^* and G4 *Terc^−/−^p53^−/−^* newborn mice. Note small body size in G4 *Terc^−/−^* mice compared with wild-type controls and that small body size is largely rescued in G4 *Terc^−/−^p53^−/−^* mice. (B) Quantification of body weight in wild-type, G4 *Terc^−/−^* and G4 *Terc^−/−^p53^−/−^* newborn mice. A total of three mice per genotype were used for the analysis (n).

It has been speculated that stem cells have a role in setting the size of organs as well as of the whole organism (see Introduction). In this regard, we have previously described that a percentage of G3 telomerase-deficient mice show a reduction in body size at the time of birth (telomerase-deficient dwarf mice), which is associated with the appearance of critically short telomeres [9]. Interestingly, both *in vivo* and *ex vivo* ESC activation defects and TPA-stimulated p53 induction are milder when G3 *Terc^−/−^* mice with standard body size are examined (Figure S1 and [9] further substantiating the link between the proliferation potential of stem cells and organismal size. Furthermore, we previously described that the dwarf phenotype of telomerase-deficient mice can be rescued by telomerase re-introduction, demonstrating that this phenotype directly provoked by the presence of critically short telomeres [9], [38].Given the above-described role for p53 in limiting the contribution of adult stem cells with dysfunctional telomeres to tissue regeneration and ESC proliferation *ex vivo*, we sought to assess whether p53 abrogation could also restore a normal body size in telomerase-deficient mice with critically short telomeres, arguing for a more global role of p53 in controlling stem cell behavior by signaling DNA damage associated to dysfunctional telomeres. To address this, we generated fourth generation (G4) *Terc^−/−^* mice, which show a 100%-penetrance of the dwarf phenotype when in a C57BL6 background (see Fig. 5a). As shown in Fig. 5a,b, genetic ablation of p53 rescued a normal body size and weight of G4 *Terc^−/−^p53^−/−^* at time of birth, which was indistinguishable to that of wild-type controls, suggesting that critically short telomeres in these mice are limiting body size. These findings argue that critically short telomeres can activate p53 during mouse development, which in turn limits the net expansion of tissues.

### p53 ablation reduces the growth arrest/senescence response in the skin and kidney of mice with critically short telomeres

**Figure 6:**
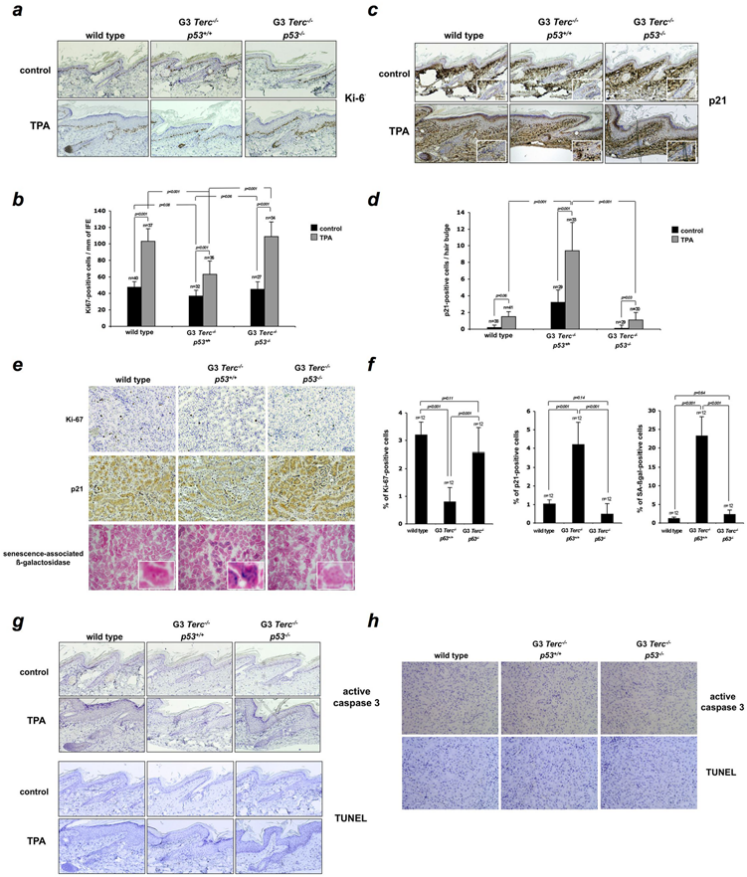
p53 loss increases the proliferation rate and ameliorates a senescence-associated response in late-generation telomerase-deficient mice without changes in apotosis rates. Representative tail-skin sections from wild-type, G3 *Terc^−/−^* and G3 *Terc^−/−^p53^−/−^* littermates stained for Ki-67 (A) and p21 (B) before and after TPA-treatment. Quantification of Ki-67-positive cells in interfollicular epidermis size (C) and p21-positive cells at the bulge region of hair follicles (D). (E) Representative kidney sections from wild-type, G3 *Terc^−/−^* and G3 *Terc^−/−^p53^−/−^* littermates stained for Ki-67, p21 and assessed for senescence-associated ß-galactosidase (SA ß-gal) activity. Inserts: high magnification images showing renal cells assessed for SA-ß-gal activity. (F) Percentage of renal cells positive for Ki-67, p21 and senescence-associated ß-galactosidase activity. n = number of sections used for quantification. (G) Representative tail-skin sections from wild-type, G3 *Terc^−/−^* and G3 *Terc^−/−^p53^−/−^* littermates stained for active caspase 3 (top) and TUNEL (bottom) before and after TPA-treatment. (H) Representative kidney sections from wild-type, G3 *Terc^−/−^* and G3 *Terc^−/−^p53^−/−^* littermates stained for active caspase 3 (top) and TUNEL (bottom).

The fact that G4 *Terc^−/−^p53^−/−^* newborns reach standard body size and weight suggests that p53 deficiency enables G4 *Terc^−/−^p53^−/−^* cells to enter into additional rounds of cell proliferation in the presence of critically short telomeres, a situation that resembles the known effects of p53 ablation in bypassing cellular senescence both *in vivo* and *in vitro* [24], [39], [40]. However, whether a p53-dependent cellular senescence and/or apoptosis response contributes to set organ and organismal size during embryonic development, or to limit stem cell functionality in adult tissues, remains unaddressed to date. To further analyze the consequences of increased p53 in mice with critically short telomeres, we examine whether tissues from telomerase-deficient mice with a reduced body size (G3 *Terc^−/−^*) enter senescence or undergo apoptosis in a p53-dependent manner. Senescence is associated with a decline in proliferation that culminates in a permanent arrest of the cell cycle [41]. Therefore, we first compared the ability of G3 *Terc^−/−^* keratinocytes to proliferate in the presence or absence of p53. As shown in Fig 6a,b, we detected slightly fewer Ki67-positive keratinocytes in G3 *Terc^−/−^p53^+/+^* untreated interfollicular (IFE) skin compared to G3 *Terc^−/−^p53^−/−^* or wild type skin, although the differences did not reach statistical significance. In contrast, a marked reduction in proliferation (Ki67-positive cells) is observed in TPA-treated G3 *Terc^−/−^p53^+/+^* skin when compared with TPA-treated G3 *Terc^−/−^p53^−/−^* and TPA-treated wild type controls (Fig. 6a,b), suggesting that the defective proliferation response to TPA of G3 *Terc^−/−^* keratinocytes is mediated by p53. In addition to growth arrest, senescence is associated at the molecular level with enhanced expression of the p53-transcriptional target p21WAF1 [41]. In resting skin conditions, a faint p21 expression was observed in G3 *Terc^−/−^p53^+/+^* epidermis, which becomes overtly visible after TPA treatment (Fig. 6c,d). Similarly to that observed for p53 expression in G3 *Terc^−/−^p53^+/+^* epidermis, positive-p21 cells located at the hair bulge and its close proximity, suggesting that the p53 transcriptionaly target p21 becomes active in stem/progenitor cells with short telomeres upon enforced proliferation. In addition, p21 expression was barely detectable at the bulge of wild type and G3 *Terc^−/−^p53^−/−^* hair follicles further (Fig. 6c,d), indicating that in the hair bulge the induction of p21 is mediated by p53. In other epidermal compartments, such as the hair bulb, infundibulum and IFE, a TPA-mediated p21 induction was observed regardless of p53 gene status (Fig. 6c,d), which suggest a minor role of p53 on the p21 upregulation in the above compartments in agreement with previous reports [42]. Next, we examined the kidney, an organ in which p53 loss partially rescues a senescence-associated phenotype induced by nuclear damage [43]. Similarly to the results obtained in keratinocytes, we detected a marked reduction in Ki67-positive cells, which was accompanied by p21-upregulation in G3 *Terc^−/−^* renal cells compared to wild types (Fig. 6e,f). In addition, we observed numerous G3 *Terc^−/−^* renal cells harboring senescence-associated ß-galactosidase activity (Fig 6e,f). Importantly, both proliferative defects and senescence markers are rescued in the absence of p53 (Fig. 6e,f), indicating the importance of p53 in mediating cellular arrest and senescence in response to short telomeres. Finally, absence of active caspase 3- and TUNEL-positive cells both in skin and kidney rules out an involvement of apoptosis in the observed phenotypes, in agreement with previously published data [9] (Fig. 6g,h). Taken together, these findings strongly suggest the presence of a p53-dependent senescence response acting on stem/progenitor cells with dysfunctional telomeres, which in turn limits their contribution to tissue size and fitness.

### Final remarks

In summary, our results define a role for the tumor suppressor p53 in monitoring ESC quality, so that ESC with dysfunctional telomeres cannot contribute to regeneration of the skin and hair, by virtue of activating a p53-dependent checkpoint. This checkpoint may be particularly important to maintain tissue fitness by ensuring that only those stem cells with a functional telomere length will contribute to tissue regeneration and tissue function. In addition, we show here that p53 ablation completely rescues a normal (indistinguishable to that of wild-type controls) organismal size at time of birth without any detectable short–term adverse effects on these mice, suggesting the existence of an early p53-mediated checkpoint controlling organismal size during embryonic development. A related early checkpoint limiting stem cell function and lifespan of mice with dysfunctional telomeres has been recently described to involve p21, a downstream target of p53 [44]. Interestingly, given the fact that p53 deletion in mice with short telomeres eventually leads to accelerated tumor formation, the premature nature of the p53-mediated blockage on stem cell activation in these mice could serve as an ahead-of-time crucial first barrier against tumor progression. Alternatively, taking into account unexpected recent findings showing little impact of DNA damage-activated p53 in cancer protection [45], [46], an intriguing possibility emerges that p53 inhibition in the case of mild DNA/telomere damage could overall resolve in beneficial effects. Finally, given that the absence of p53 seems to exert a general effect on mice, as indicated by the fact that the small body size phenotype is completely rescued in G4 *Terc^−/−^p53^−/−^* mice, these results suggest that p53 abrogation could conceivably restitute stem cells functionality in other organs harboring short telomeres.

## Methods

### Animals and treatment regimens

*Terc^+/−^* and *p53^+/−^* mice were first intercrossed to generate *Terc^+/−^p53^+/−^* double heterozygous mice and then mated to generate first generation (G1) *Terc^−/−^p53^+/−^* littermates. G1 *Terc^−/−^p53^+/−^* littermates were interbreed for successive generations to obtain late generation G3–G4 *Terc^−/−^p53^−/−^* double mutant mice as well as their G3–G4 *Terc^−/−^* littermate controls [25]. The genetic background for all genotypes was a pure C57BL6 background.

To induce LRC mobilization, IFE hyperplasia and hair growth, tail skin from a group of three 71-days-old mice per genotype in the resting phase of the hair cycle was topically treated every 48 hours with TPA (20 nmol in acetone) for four doses with the exception of the experiments represented in Fig. 2, Fig. 6 and Figure S1a (six doses). Three control mice of each genotype were treated with acetone alone. 24-hours after the last TPA treatment skin from a group of three 71-days-old mice per genotype were sacrificed and the tail skin analyzed.

All animal experiments and husbandry were carried out in accordance with guidelines from Federation of European Laboratory Animal Science Association (FELASA).

### Wound-healing experiments

Three full-thickness punch biopsies extending through the epidermis and dermis (punch diameter 4 mm; PFM, Köln, Germany) were performed in three wild-type, three G3 *Terc^−/−^p53^−/−^* and three G3 *Terc^−/−^p53^+/+^* six K5-mTERT mice (2 months of age) after depilation. Mice were anesthetized prior to wound creation. The wound-healing rate was calculated as the percentage of initial wound area with time. Wound areas were calculated with the formula (area = *r* ^2^; where *r* is the ratio of the wound).

### Histology and immunohistochemistry of skin

Tail skin samples were harvested from mice and fixed overnight in neutral-buffered formalin at 4°C, dehydrated through graded alcohols and xylene, and embedded in paraffin. For determination of IFE thickness and HF length, dissected skin was cut parallel to the spine and sections were cut perpendicular to the skin surface in order to obtain longitudinal HF sections. 5 µM sections were used for hematoxylin-eosin staining and immunohistochemistry (IHC). Prior to IHC, slides were de-paraffinized, re-hydrated, immersed in 10 mM citrate solution and epitopesretrieved by three high-power, 5 min microwave pulses. Slides were washed in water, blocked in 1∶10 dilution of normal goat serum (Vector Labs) and incubated with primary antibodies: p53 at 1∶150 (CM5p, Novacastra), CD34 at 1∶25 (MEC14.7, Abcam), keratin 15 at 1∶25 (LHK15, NeoMarkers), BrdU at 1∶35 (BU-1, Amersham), Ki-67 at 1∶200 (SP6, Master Diagnostica), p21 at 1∶250 (C-19G; Santa Cruz Biotechnology) and active caspase-3 at 1∶150 (R&D Systems). Slides were then incubated with secondary biotinylated antibodies from Vector labs (goat anti-rabbit at 1∶200 or goat anti-mouse at 1∶200), followed by signal development with an immunoperoxidase reagent (ABC-HRP, Vector Labs) and DAB (Sigma) as the substrate. The TUNEL assay was performed using the Apoptag Kit manufactured by Chemicon. Sections were lightly counterstained with hematoxylin and analyzed by light microscopy.

### LRC detection

To detect LRCs in whole-mounts, fixed epidermal sheets were blocked and permeabilized by incubation in a modified PB buffer [32] containing 0.5% BSA and 0.5% Triton X-100 in TBS for 30 minutes. Subsequently, epidermal sheets were immersed for 30 min in 2 M HCL at 37°C, incubated overnight with a mouse anti- BrdU antibody conjugated with fluorescein (Roche) at 1∶50 in modified PB buffer, washed four times in PBS containing 0.2% Tween 20, and mounted in Vectashield (Vector Labs). Tissues were then washed as previously described and cover-slipped with Vectashield with DAPI (Vector Labs).

### Colony forming assay

One thousand mouse keratinocytes per genotype isolated from neonatal skin were seeded onto mitomycin C (10 µg/ml, 2 hours) treated J2-3T3 fibroblast (105 per well, 6 well dishes) and grown at 37°C/5% CO2 in Cnt-02 medium (CELLnTEC Advanced Cell Systems AG, Bern, Switzerland). After one week of cultivation, dishes were rinsed twice with PBS, fixed in 10% formaldehyde and then stained with 1% Rhodamine B to visualizy colony formation. Colony size and number were measured using three dishes per experiment, over a total of three separate experiments.

### Statistical analysis

Statistical analysis of differences between different mouse cohorts was performed using Student t test with “two tails” and “two-samples of equal variance”. Microsoft Excel v.X was used for calculations.

## Supporting Information

Figure S1 LRC activation and TPA-mediated p53 induction in G3 Terc−/− mice with distinct body sizes. (A) Representative images of tail-sections from G3 Terc−/− with a “small” body size and a “standard” body size stained for p53 before and after TPA-treatment. (B) Quantification of p53-positive cells that locate to the hair follicle (left panel) and to the interfollicular epidermis (right panel). (C) Representative confocal micrographs of tail follicles from G3 Terc−/− mice with a “small” body size and a “standard” body size stained for BrdU (green) before (control) and after (TPA) TPA treatment. (D) Number of LRCs in the conditions shown in (C).
(7.09 MB TIF)

Methods S1 (0.05 MB DOC)
