# Supplementary figures and images for: A p53-Dependent Response Limits Epidermal Stem Cell Functionality and Organismal Size in Mice with Short Telomeres
Source: PLoS One. 2009 Mar 19;4(3):e4934. doi: 10.1371/journal.pone.0004934 (PMC2654505; doi:10.1371/journal.pone.0004934)

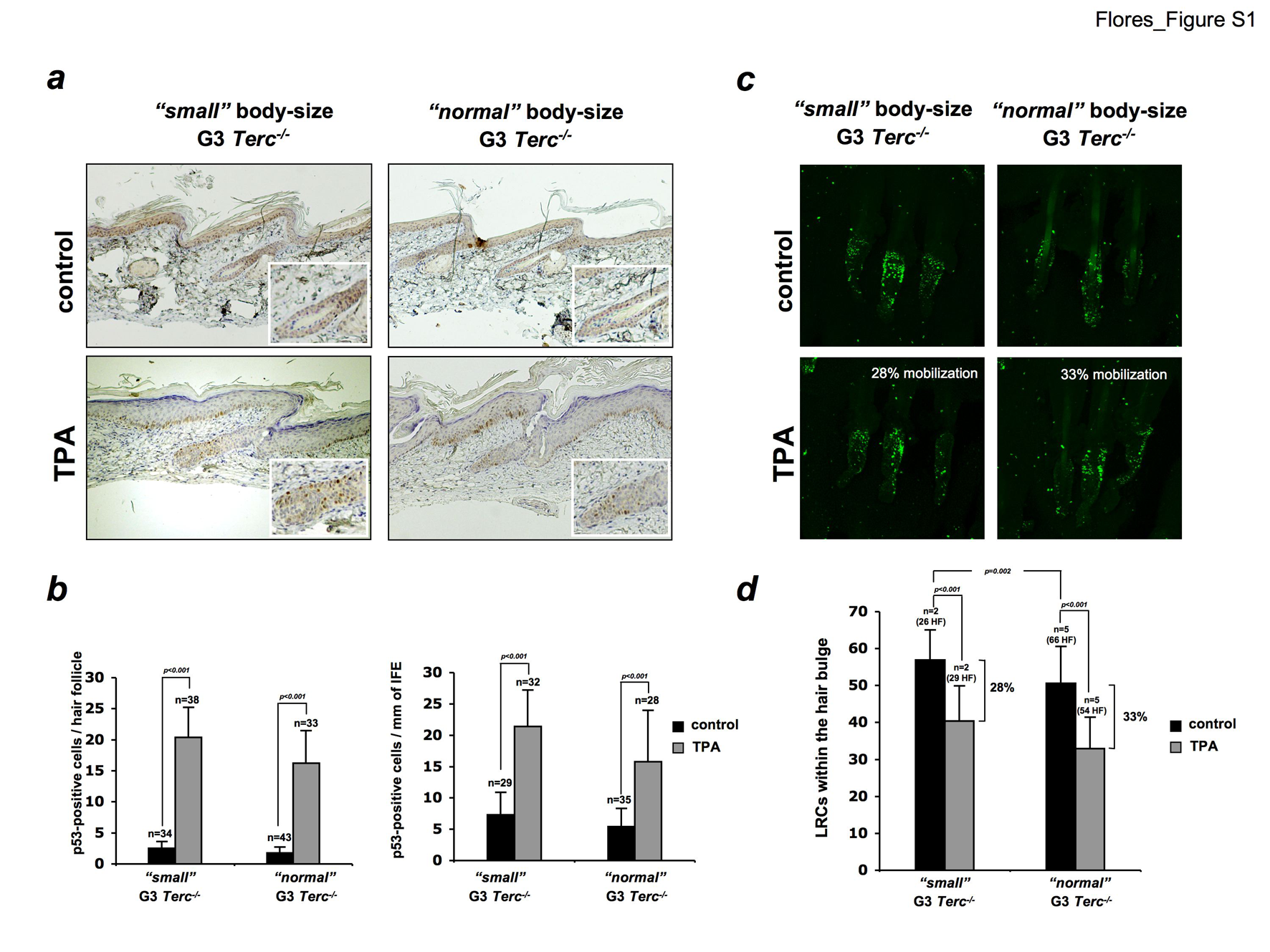

Supplement: Figure S1 — LRC activation and TPA-mediated p53 induction in G3 Terc−/− mice with distinct body sizes. (A) Representative images of tail-sections from G3 Terc−/− with a “small” body size and a “standard” body size stained for p53 before and after TPA-treatment. (B) Quantification of p53-positive cells that locate to the hair follicle (left panel) and to the interfollicular epidermis (right panel). (C) Representative confocal micrographs of tail follicles from G3 Terc−/− mice with a “small” body size and a “standard” body size stained for BrdU (green) before (control) and after (TPA) TPA treatment. (D) Number of LRCs in the conditions shown in (C). (7.09 MB TIF) [file pone.0004934.s001.tif]
