# Supplementary material for: A p53-Dependent Response Limits Epidermal Stem Cell Functionality and Organismal Size in Mice with Short Telomeres
Source: PLoS One. 2009 Mar 19;4(3):e4934. doi: 10.1371/journal.pone.0004934 (PMC2654505; doi:10.1371/journal.pone.0004934)
Supplement: Methods S1 — (0.05 MB DOC) [file pone.0004934.s002.doc]

**Supplementary Methods**

**Labeling of LRCs**

LRC were obtained basically as described (Bickenbach et al. 1986; Cotsarelis et al. 1990; Braun et al. 2003) with some modifications. Briefly, groups of 6 neonatal mice per genotype were injected with 50 mg/kg of body weight BrdU (Sigma) diluted in PBS. Each animal received a daily injection beginning at day 4 of life for a total of 5 days. After the labeling period, mice were allowed to grow for 60 days before the initiation of any treatment. Cells retaining the label at the end of the treatment were identified as label-retaining cells (LRCs).

**Preparation of whole-mounts**

Whole-mounts of mouse tail epidermis were prepared as previously described (Braun et al. 2003). Briefly, after mice were sacrificed with CO2 and tails amputated, skin was peeled from the tails and incubated in 5 mM EDTA in PBS at 37ºC for 4 hours. Using forceps, intact sheets of epidermis were separated from the dermis and fixed in neutral-buffered formalin for 2 hours at room temperature. Fixed epidermal sheets were maintained in PBS containing 0.2% sodium azide at 4ºC prior to labeling.

**Confocal microscopy**

A laser scanning confocal microscope (LEICA TCS-SP2-AOBS) was used to obtain fluorescence images. Image stacks of 60-80 µm were obtained through the z dimension, at steps 1.0 µm apart, using a PL APO 20x/0.70 PH2 as lens. Maximum intensity projections of the image stacks were then generated using Leica LCS Software.

**Isolation of newborn keratinocytes**

2 days old mice were sacrificed, soaked in Betadine (5 min), in a PBS antibiotics solution (5 min), in 70% ethanol (5 min), and in a PBS antibiotics solution (5 min). Limbs and tail were amputated, and the skin peeled off using forceps. Skins were then soaked in PBS (2 min), PBS antibiotics solution (2 min), 70% ethanol (1 min) and in PBS antibiotics solution (2 min). Using forceps, each skin was floated on the surface of 1x trypsin (Sigma) solution (4ml on 60mm cell culture plate) for 16 h at 4ºC. Skins were transferred to a sterile surface, and the epidermis separated from the dermis using forceps, minced and stirred at 37^o^C for 30 min in serum-free Cnt-02 medium (CELLnTEC Advanced Cell Systems AG, Bern, Switzerland). The cell suspension was filtered through a sterile teflon mesh (Cell Strainer 0.7 m, Falcon) to remove cornified sheets. Keratinocytes were then collected by 5 mincentrifugation (160 g) for 10 min and seeded on collagen I pre-coated cell culture plates (BD Biosciences).
